# The Potential Use of *Wolbachia*-Based Mosquito Biocontrol Strategies for Japanese Encephalitis

**DOI:** 10.1371/journal.pntd.0003576

**Published:** 2015-06-18

**Authors:** Claire L. Jeffries, Thomas Walker

**Affiliations:** Department of Disease Control, London School of Hygiene and Tropical Medicine, London, United Kingdom; Centers for Disease Control and Prevention, Puerto Rico, UNITED STATES

## Abstract

Japanese encephalitis virus (JEV) is a zoonotic pathogen transmitted by the infectious bite of *Culex* mosquitoes. The virus causes the development of the disease Japanese encephalitis (JE) in a small proportion of those infected, predominantly affecting children in eastern and southern Asia. Annual JE incidence estimates range from 50,000–175,000, with 25%–30% of cases resulting in mortality. It is estimated that 3 billion people live in countries in which JEV is endemic. The virus exists in an enzootic transmission cycle, with mosquitoes transmitting JEV between birds as reservoir hosts and pigs as amplifying hosts. Zoonotic infection occurs as a result of spillover events from the main transmission cycle. The reservoir avian hosts include cattle egrets, pond herons, and other species of water birds belonging to the family *Ardeidae*. Irrigated rice fields provide an ideal breeding ground for mosquitoes and attract migratory birds, maintaining the transmission of JEV. Although multiple vaccines have been developed for JEV, they are expensive and require multiple doses to maintain efficacy and immunity. As humans are a “dead-end” host for the virus, vaccination of the human population is unlikely to result in eradication. Therefore, vector control of the principal mosquito vector, *Culex tritaeniorhynchus*, represents a more promising strategy for reducing transmission. Current vector control strategies include intermittent irrigation of rice fields and space spraying of insecticides during outbreaks. However, *Cx*. *Tritaeniorhynchus* is subject to heavy exposure to pesticides in rice fields, and as a result, insecticide resistance has developed. In recent years, significant advancements have been made in the potential use of the bacterial endosymbiont *Wolbachia* for mosquito biocontrol. The successful transinfection of *Wolbachia* strains from *Drosophila* flies to *Aedes* (*Stegomyia*) mosquitoes has resulted in the generation of “dengue-refractory” mosquito lines. The successful establishment of *Wolbachia* in wild *Aedes aegypti* populations has recently been demonstrated, and open releases in dengue-endemic countries are ongoing. This review outlines the current control methods for JEV in addition to highlighting the potential use of *Wolbachia*-based biocontrol strategies to impact transmission. JEV and dengue virus are both members of the *Flavivirus* genus, and the successful establishment of *Drosophila Wolbachia* strains in *Cx*. *Tritaeniorhynchus*, as the principal vector of JEV, is predicted to significantly impact JEV transmission.

## Methods

This review was prepared by searching the literature, including publication databases such as PubMed and Web of Science, on current methods used for vector control of Japanese encephalitis virus (JEV) and by providing an up-to-date review of *Wolbachia*-based biocontrol strategies for dengue and how these strategies could target Japanese encephalitis (JE).

## Introduction

**Fig 1:**
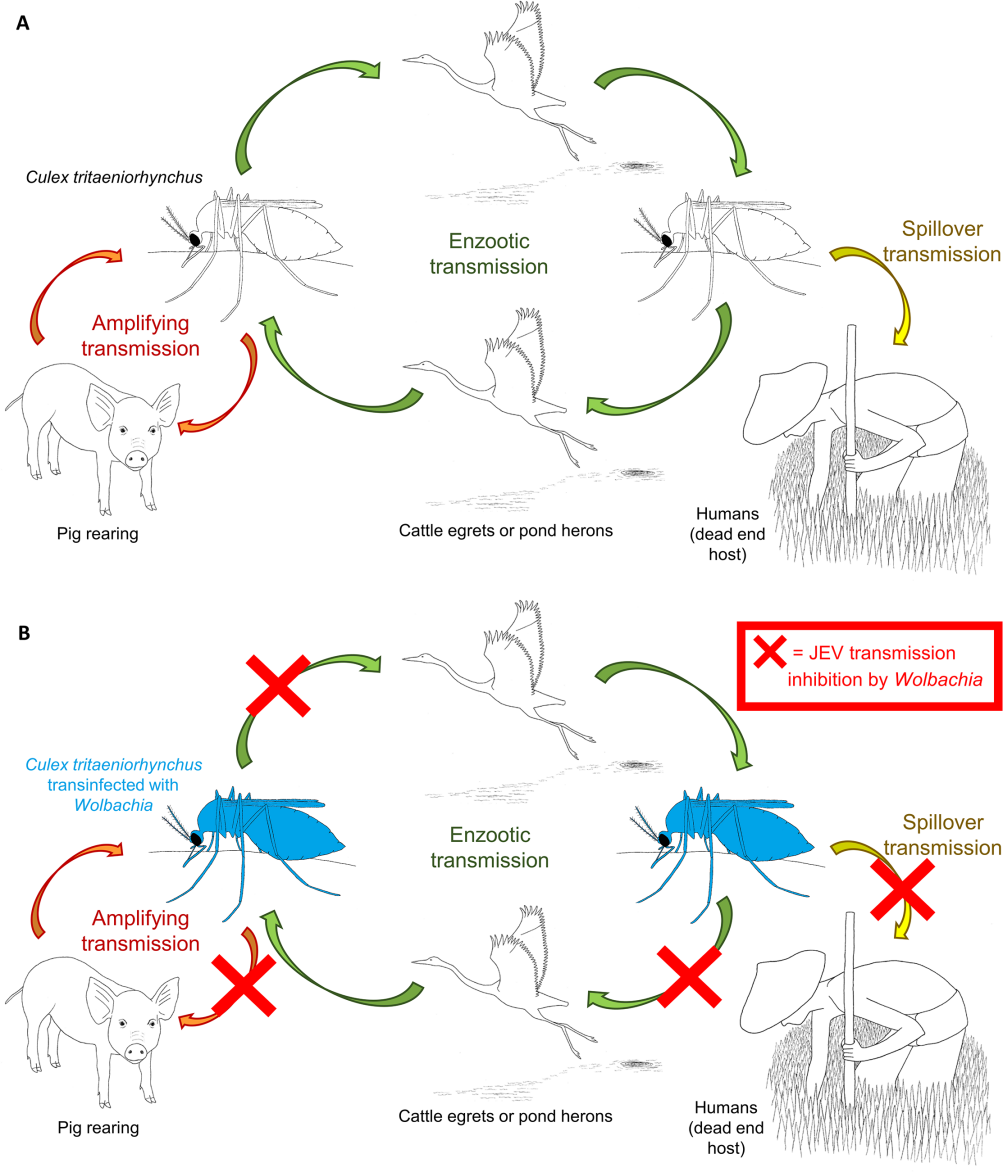
JEV transmission cycle and potential inhibition through the introduction of *Wolbachia*-infected *Cx*. *tritaeniorhynchus* mosquitoes. (A) The enzootic sylvatic cycle is maintained by reservoir bird hosts (cattle egrets and pond herons) in close association with *Cx*. *tritaeniorhynchus* mosquitoes in rice fields. JEV is amplified in pigs, and zoonotic infection occurs as a result of spillover events, but human-to-human transmission is not known to occur at significant levels. (B) The JEV transmission cycle could be interrupted at various points using a *Wolbachia*-based biocontrol strategy in which JEV-refractory mosquitoes are unable to maintain the enzootic transmission cycles or transmit the virus to humans.

Numerous medically important arthropod-borne viruses (arboviruses) are transmitted to humans through the bites of infected mosquitoes. JEV is a neurotropic flavivirus transmitted primarily by *Culex tritaeniorhynchus* mosquitoes. The resulting disease, JE, is now endemic in large parts of Asia and the Pacific, with over 3 billion people at risk of infection [1,2]. It is estimated that less than 1% of human JEV infections result in encephalitic disease [3]. However, viral encephalitis caused by JEV can lead to fever, coma, seizures, paralysis, and death. JE is predominantly a disease of children in eastern and southern Asia, with annual incidence estimates ranging from 50,000–175,000 cases and 25%–30% of encephalitis cases resulting in mortality [3]. A further 30%–50% of JE survivors suffer serious neurological, cognitive, or psychiatric sequelae [4]. JEV exists in an enzootic transmission cycle among mosquitoes and domestic pigs, with the reservoir sylvatic bird hosts being primarily water birds from the *Ardeidae* family, including cattle egrets and pond herons (Fig 1A) [1]. As JEV-infected pigs act as amplifying hosts, domestic pig rearing is an important risk factor for human transmission. Irrigated rice fields provide an ideal breeding ground for *Cx*. *tritaeniorhynchus* and attract migratory birds, maintaining sylvatic transmission.

The geographic transmission zone extends from the China–Russia border region in the north to northern Australia in the south and from the Western Pacific islands in the east to the India–Pakistan border region in the west [5]. The epidemiology of JE can be divided into two distinct patterns based on climate. In temperate countries such as China and Japan, seasonal outbreaks are correlated with increased temperatures and rainfall in summer months. In contrast, tropical and subtropical countries in Southeast Asia have sporadic JE cases throughout the year, with reports peaking during the rainy season. The annual incidence of disease in Japan and Korea has declined because of improved living standards and vaccination programs [2]. However, the number of reported cases of human disease has increased in developing countries such as Bangladesh, Pakistan, and Indonesia because of increased population growth, intensified rice cultivation, and pig rearing. JEV also emerged in the Torres Strait Islands and spread to the far north of Australia in the late 1990s [6], highlighting the possibility that the virus could become established in other parts of the world. Transmission of JEV was traditionally considered to be mainly limited to rural areas, where the presence of mosquito vectors in rice fields coincides with pork production. However, several studies in urban areas have detected virus within the local mosquito vector populations, and seroconversion in urban vertebrate hosts (including humans) has been reported [7]. The human population has increased significantly within the past 50 years in both JEV-endemic regions and in areas where epidemics occur, with increasing trends in urbanization and an associated likely increase in urban agriculture. Therefore, the possibility that JEV transmission can occur in urban areas with the presence of vectors and a much greater human population density, combined with the potential for an increase in urban livestock, has the potential to significantly increase the number of cases in the future [7].

## JEV and Mosquito Vectors

**Fig 2:**
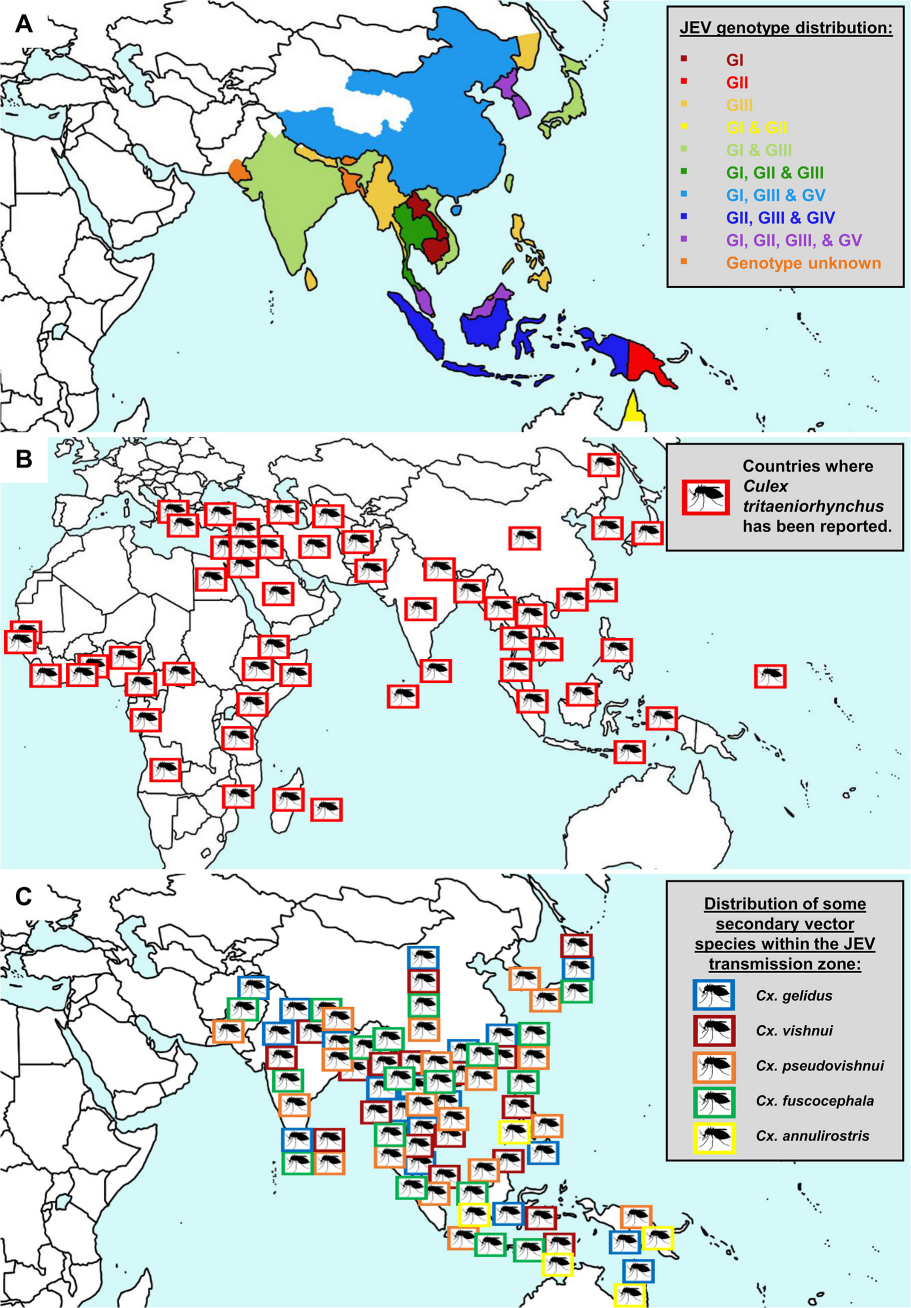
JEV genotypes, the geographical range of *Cx*. *tritaeniorhynchus* and secondary vectors. (A) The five recognized genotypes of JEV (I–V) are shown in regions where the genotype (G) has been confirmed to be responsible for JE epidemics. (B) The areas in which the principal mosquito vector, *Cx*. *tritaeniorhynchus*, has been documented highlight the wide geographical range of this species of mosquito. (C) The distribution of some of the secondary vector species is shown within the JEV transmission zone.

JEV is within the genus *Flavivirus*, which contains more than 70 enveloped viruses, including other medically important arboviruses such as dengue virus (DENV), West Nile virus (WNV), and yellow fever virus (YFV) [8]. Although first isolated in Japan in 1935, JEV appears to have evolved from its ancestral form to the present genotypic forms in Southeast Asia, over a relatively short period [9]. Phylogenetic studies have classified JEV into five geographically and epidemiologically distinct genotypes: GI to GV [9]. GIII had been the predominant genotype responsible for JE epidemics until the 1990s. However, studies have shown that GI is displacing GIII in many regions and has become the dominant genotype [10,11]. The emergence of GI throughout Asia is likely the result of viral, environmental, and host factors [12]. The principal vector of JEV is *Cx*. *tritaeniorhynchus*, which has a wide distribution including parts of Africa, the Middle East, and southern Europe in addition to the JE-endemic areas of Asia. A recent outbreak in China in 2013, resulting in 407 confirmed cases, was attributed to high JEV infection rates in *Cx*. *tritaeniorhynchus* (9.1 per 1,000 mosquitoes) using a maximum likelihood estimation [13]. The presence of this species was recently documented in parts of western Greece [14], highlighting the potential risk of JEV transmission in nonendemic areas outside of Asia. The principal vector of JEV in Australia is *Cx*. *annulirostris* [15], and other vector species such as *Cx*. *Gelidus*, *Cx*. *Vishnui*, *Cx*. *Pseudovishnui*, and *Cx*. *fuscocephala* have been implicated as secondary or regional vectors in certain endemic areas [16]. Fig 2 highlights the presence of JEV genotypes identified in JE-endemic areas, the worldwide geographical range of the principal mosquito vector *Cx*. *Tritaeniorhynchus*, and the distribution of some of the secondary vector species within the JEV transmission zone. The control of JEV has focused on vaccines and mosquito vector control (Table 1), as there are still no specific drug treatments available for infected patients.

**Table 1:** Current and potential strategies for controlling Japanese encephalitis and the problems associated with each control method.

Control Strategy	Difficulties for Implementation
Human vaccination	Expensive, multiple doses required, low rates in rural areas
Pig vaccination	Expensive, maternal antibodies reduce efficacy
Insecticide space spraying	Resistance from exposure to pesticides in rice fields, logistically difficult on a large scale
Indoor residual insecticide spraying	*Cx*. *tritaeniorhynchus* is exophilic
Intermittent irrigation	Logistically difficult in large areas with inadequate infrastructure
Genetically modified mosquitoes	Genetic modification of *Cx*. *tritaeniorhynchus* required, cost-effectiveness and implementation over large areas
*Wolbachia*-infected mosquitoes	*Wolbachia* transinfection of *Cx*. *tritaeniorhynchus* required: long-term effectiveness unknown

## JE Vaccination

The mouse brain-derived killed-inactivated JE-VAX was the only commercially available vaccine worldwide for several decades despite adverse effects, high production costs, and the need for 2–3 primary doses plus boosters [1,17]. Significant allergic and neurological side effects led to a halt in production of JE-VAX in 2006 [18,19]. A live-attenuated cell culture-derived JE vaccine, SA14-14-2, was developed in China in 1988 and has been administered to Chinese children since production commenced. SA14-14-2 is generated in primary hamster kidney (PHK) cells, and concerns with the quality control of production have prevented its application expanding in order to become an internationally available vaccine [20]. PHK-derived inactivated vaccines have been further adapted to be produced in African green monkey kidney (Vero) cells [21]. One vaccine (designated IC51) has been licensed since 2009 for use in countries including the United States, Europe, Australia, and India under various trade names, including IXIARO [22]. However, as there are concerns with this, the only WHO-recommended vaccine, because of side effects [23], additional vaccines are in various stages of development. A recombinant, live-attenuated vaccine based on a chimeric yellow fever/Japanese encephalitis virus (ChimeriVax-JE) was developed recently [24] and is now commercially available in Australia and Thailand. Although multiple vaccines have been developed for JEV, they are expensive and require multiple doses to maintain efficacy and immunity [25]. In addition, all registered vaccines are derived from GIII [11], which may be problematic given the replacement of GIII with GI as the dominant genotype. In most JEV-endemic rural settings, vaccination rates are often low, and vaccines are unlikely to result in eradication given humans are predominantly “dead-end” hosts, in that viremia is insufficient for onward transmission (Fig 1A). The potential of vaccinating pigs has also been explored, but the majority are slaughtered at 6–8 months. This means annual vaccination of piglets would be required, and the presence of maternal antibodies prevents the live-attenuated vaccine being effective in pigs less than 6 months of age. Therefore, there are too many limitations for this strategy to be effectively implemented [26]. As a result, mosquito vector control strategies represent a method more likely to eradicate JEV transmission than vaccination of humans or pigs, as mosquitoes are also responsible for maintaining transmission in reservoir bird hosts in the sylvatic cycle.

## Current Vector Control and the Need for Novel Strategies

Vector control for JEV has predominantly been focused on environmental management of rice fields. Alternative wetting and drying of rice fields (intermittent irrigation) has shown success in reducing mosquito populations [27]. However, there are significant logistical difficulties with intermittent irrigation, including the requirement to apply this method to all rice fields over large areas [28], which is not possible with inadequate infrastructure. The use of insecticides (pyrethroids, organophosphates, and carbamates) has been limited for JEV vector control, although space spraying to target adult mosquitoes has been undertaken during outbreaks of JE in densely populated areas. However, the heavy use of pesticides in rice fields has led to significant levels of insecticide resistance in mosquitoes [29]. Logistical difficulties in employing large-scale insecticide treatment of rice fields, often in isolated rural villages, are also problematic for vector control of JEV. Indoor residual spraying using dichlorodiphenyltrichloroethane (DDT) and other chemicals has been ineffective in reducing JEV transmission, as *Cx*. *tritaeniorhynchus* is largely exophilic, resting outdoors [30].

Although climate change could further increase the geographical range of JEV transmission, in a similar way as predicted for DENV [31,32], the potential impact is yet to be determined. It is predicted that climate change will lead to increases in mosquito vector density, incursion of exotic mosquito species into novel areas, changes in agricultural practices, and migration of host reservoir birds. In particular, rice fields in JEV-endemic areas would likely become more arid and the subsequent increase in flooding, either through irrigation or extreme weather events, would provide optimal breeding conditions for *Cx*. *tritaeniorhynchus* [2]. Rapid outbreaks of JE are difficult to control, with traditional methods such as space spraying of insecticides having little impact because of the unpredictability and infrequency of outbreaks. Climate change may also influence migration patterns of birds, which may result in long-distance JEV dissemination in new areas. WNV is a closely related zoonotic flavivirus that has a similar enzootic transmission cycle with reservoir migratory birds. The introduction of the closely related WNV to novel areas has been strongly associated with bird migration [33,34], and climate change is likely to influence WNV outbreaks [35,36]. As there is very little known about the particular migration patterns of the avian reservoirs for JEV, the likely impact of climate change remains unknown [37]. In recent years, significant advances have been made in the potential use of the bacterial endosymbiont *Wolbachia* for mosquito biocontrol. This has included the successful transinfection of *Cx*. *quinquefasciatus* to create a *w*Pip strain variant superinfection [38]. *Wolbachia* transinfection of *Cx*. *tritaeniorhynchus* could provide the basis for an environmentally friendly and cost-effective biocontrol strategy that could significantly impact JEV transmission (Box 1), which is likely to remain applicable even if JEV-endemic regions expand in the future as a result of climate change.

## *Wolbachia*-Based Mosquito Biocontrol Strategies

*Wolbachia pipientis* are maternally inherited alphaproteobacteria that live intracellularly in over 60% of all insect species [39]. *Wolbachia* were initially identified in the ovaries of *Cx*. *pipiens* mosquitoes, and these endosymbionts manipulate host reproduction to enhance their own transmission. In mosquitoes, *Wolbachia* induces a phenotype known as cytoplasmic incompatibility (CI), which results in the generation of unviable offspring when an uninfected female mates with a *Wolbachia*-infected male. In contrast, *Wolbachia*-infected females can produce viable progeny when they mate with both infected and uninfected males, resulting in a selective reproductive advantage over uninfected females. The CI phenotype allows the maternally transmitted *Wolbachia* to efficiently invade host populations without being infectious or moving horizontally between individuals [40].

During the 1970s and 1980s, several studies examined the effect of CI and the potential for application of the phenotype for vector control, in addition to elucidating the role of *Wolbachia* in producing CI in insect populations [41,42]. The discovery in the late 1990s of the virulent *w*MelPop strain in *Drosophila melanogaster* flies, which dramatically lowered the lifespan of its host [43], led to the idea that *Wolbachia* could also be used to manipulate insect longevity to reduce pathogen transmission. Mosquito-borne pathogens such as JEV require a significant extrinsic incubation period (EIP) in the female mosquito after uptake in an infectious blood meal before the pathogen migrates to the salivary glands to be transmitted to a host. For JEV the EIP is believed to be between 7 and 14 days, but it has been found to vary from 6 to 20 days dependent on temperature [16]. The *w*MelPop strain was proposed as having the potential to shorten the longevity of adult female mosquitoes so that the majority of females die before the EIP has elapsed [44,45]. More recently, several strains of avirulent *Wolbachia* were found to protect their native *Drosophila* hosts against infection by pathogenic RNA viruses [46,47]. Interestingly, major mosquito arboviral vectors such as *Aedes (Stegomyia) aegypti* and *Cx*. *tritaeniorhynchus* do not harbor natural *Wolbachia* infections. However, the successful establishment of *Wolbachia* strains in *Ae*. *aegypti* has now been accomplished, and the phenotypic effects suggest *Wolbachia* could significantly reduce arboviral transmission in naïve mosquito hosts.

## *Wolbachia* and Dengue Vector Competence in *Ae*. *aegypti* Mosquitoes

**Fig 3:**
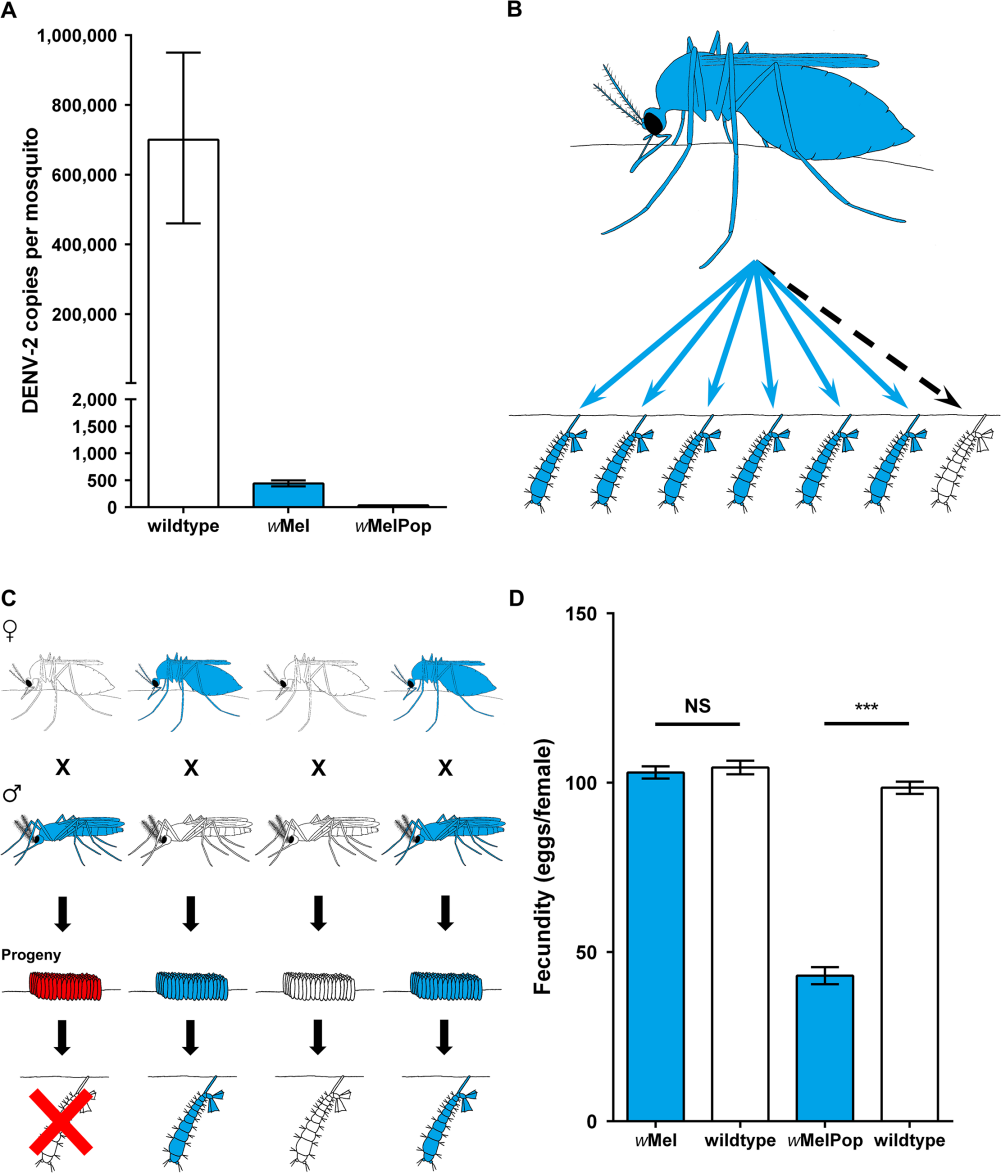
*Wolbachia* phenotypic effects for mosquito biocontrol. (A) Pathogen inhibition of DENV in *Ae*. *aegypti* by transinfected *Drosophila Wolbachia* strains significantly decreases the DENV infection levels in mosquitoes 14 days after an infectious blood meal. (B) Maternal transmission of *Wolbachia* from female mosquitoes to progeny is close to 100% for transinfected *Drosophila* strains in mosquitoes. (C) *Wolbachia*-induced reproductive phenotype cytoplasmic incompatibility in mosquitoes allowing rapid invasion of uninfected mosquito populations. (D) Fitness costs (e.g., fecundity) of transinfected *Drosophila Wolbachia* strains to *Ae*. *aegypti*, which impact the ability of some strains to invade mosquito populations. (A) and (D) are adapted from [50].

The first successful transinfection of *Ae*. *aegypti* used the *w*AlbB strain of *Wolbachia* from closely related *Ae*. *albopictus* mosquitoes [48]. Transinfection of *Drosophila Wolbachia* strains *w*MelPop-CLA and *w*Mel was accomplished by first maintaining the bacteria in mosquito cell lines [49,50]. All three transinfected *Wolbachia* strains significantly reduce the vector competence of *Ae*. *aegypti* for DENV under laboratory conditions [50–52]. Both total (Fig 3A) and disseminated DENV is significantly reduced in *Drosophila Wolbachia–*infected mosquitoes. Furthermore, the presence of infectious DENV in mosquito saliva was not observed for *Wolbachia*-infected mosquitoes [50]. The *w*Mel strain was also shown to result in complete blockage of DENV transmission in transinfected *Ae*. *albopictus* [53].

## *Wolbachia* Invasion of Wild Mosquito Populations

The applied use of *Wolbachia* strains to impact arboviral transmission requires invasion of wild mosquito populations. *Wolbachia*-infected females must vertically transmit the bacteria to their progeny at a high frequency (Fig 3B), and then CI (Fig 3C) can spread the infection. *Wolbachia* strains in *Ae*. *aegypti* show maternal transmission rates close to 100% and induce CI [48,50,54]. However, *Wolbachia*-infected mosquitoes can only spread and invade uninfected populations if the fitness costs such as fecundity (Fig 3D) to the mosquito are less than the fitness advantage that CI provides. *Ae*. *aegypti* mosquitoes infected with the virulent *w*MelPop-CLA strain are subject to greater fitness costs than mosquitoes infected with the avirulent *w*Mel strain [50]. These significant fitness costs of the *w*MelPop-CLA strain were predicted to inhibit invasion of wild mosquito populations [55]. The invasive potential of the two *Drosophila Wolbachia* strains in *Ae*. *aegypti* was tested in large purpose-built semifield cages in northern Australia [56]. The results of these semifield experiments revealed that the *w*Mel strain is likely to be the most successful at invading wild mosquito populations [50]. *Ae*. *aegypti* mosquitoes infected with the *w*Mel strain were recently introduced into the wild through open releases in two locations near Cairns, Australia, after obtaining the necessary regulatory approval [57]. The *w*Mel strain successfully invaded these two natural mosquito populations, reaching near-fixation in a few months following releases. After more than 2 years in the field, the infection has continued to demonstrate complete CI with minimal deleterious fitness effects. Although a low frequency of uninfected mosquitoes was detected, it would appear this was due to a low number of immigrants, and the infection appears to be stable under field conditions [58]. The persistence of an inhibitory effect on arboviral replication within wild *Wolbachia*-infected mosquitoes will be key to the success of any release program. Vector competence assays with field *w*Mel-infected *Ae*. *aegypti* mosquitoes, collected 1 year following field release, indicated very low levels of DENV replication and dissemination [59]. The level of viral interference was similar in outcrossed laboratory lines and field-collected mosquitoes. However, the density of *Wolbachia* increased following blood feeding to a greater extent in field mosquitoes compared to laboratory colonies [59]. As *Wolbachia* density is correlated to viral interference in both native *Drosophila* [60] and transinfected *Ae*. *Aegypti* [50] hosts, repeated blood feeding on human hosts may increase the viral blocking phenotype in field mosquito populations.

## JE as a Potential Target for *Wolbachia*-Based Biocontrol Strategies

There are several lines of evidence that would suggest that JE could be targeted using *Wolbachia*-based biocontrol. Firstly, JEV is part of the same genus as DENV (*Flavivirus)*, so there is a reasonable expectation that *Wolbachia* strains would provide similar inhibitory effects in transinfected mosquitoes. In laboratory experiments, *Wolbachia* inhibits the replication of multiple DENV serotypes with similar efficacy [59]. Several studies have also shown that *Wolbachia* has a wide range of inhibitory effects on other mosquito-borne human pathogens when transinfected into naïve mosquito species (Table 2). For example, *Drosophila Wolbachia* strains also significantly inhibit the replication of Chikungunya virus (CHIKV) in *Ae*. *aegypti* [61]. Pathogen inhibition by transinfected *Wolbachia* strains also occurs for filarial nematodes [62] and malaria parasites [63,64]. The mechanism underlying viral interference is not fully known, but the density of *Wolbachia* strains in particular insect tissues influences the extent of viral interference [50]. Several mechanisms have been postulated for *Wolbachia*-mediated antiviral activity, including direct competition for space or cellular resources and effects on various immune signaling pathways; however, further investigation is required [65]. *Drosophila Wolbachia* strains grow to high densities in their native and transinfected hosts and provide strong inhibition of both insect viruses in *Drosophila* [46] and DENV in mosquitoes [50]. Therefore, successful establishment of *Drosophila Wolbachia* strains in *Cx*. *tritaeniorhynchus* is reasonably expected to have a significant impact on JEV transmission.

**Table 2:** List of mosquito vector species infected with native or transinfected *Wolbachia* strains and their relative inhibitory effect on vector competence of arboviruses.

Mosquito Species	*Wolbachia* Strain (Native or Transinfected)	Arbovirus	Inhibitory Effect on Vector Competence	References
*Ae*. *aegypti*	*w*AlbB (transinfected)	DENV	++	[48,52]
	*w*Mel (transinfected)	DENV	++	[50,59]
		CHIKV	+	[61]
	*w*MelPop (transinfected)	DENV	+++	[50,51,54]
		CHIKV	+++	[61]
*Ae*. *albopictus*	*w*AlbA and *w*AlbB (native)	DENV	+	[66]
	wMel (transinfected)	DENV	++	[53]
		CHIKV	++	[67]
*Ae*. *polynesiensis*	*w*PolA (native)	DENV	-	[68]
	*w*AlbB (transinfected)	DENV	++	[68]
*Cx*. *pipiens*	*w*Pip (native)	WNV	+	[69]

Secondly, *Cx*. *tritaeniorhynchus* does not harbor a natural *Wolbachia* infection [70] and is responsible for the majority of JEV transmission. Although there are additional secondary vectors in Asia and *Cx*. *annulirostris* is responsible for the limited JEV transmission in northern Australia [15], the replacement of wild *Cx*. *tritaeniorhynchus* with JEV-refractory populations would likely have significant impacts on both virus transmission and human cases of disease. There is also the potential for transinfection of other secondary/regional vector species of importance if *Wolbachia* biocontrol in *Cx*. *tritaeniorhynchus* had brought eradication within reach. Other species of *Culex* mosquitoes responsible for human disease transmission are infected with native strains of *Wolbachia*. Indeed, the difference in vector competence of several *Culex* species may be due to the presence of these resident *Wolbachia* strains. For example, *Cx*. *quinquefasciatus* is infected with the *w*Pip strain of *Wolbachia* and is generally less susceptible to WNV than *Cx*. *tarsalis* [71], which is not infected with *Wolbachia*. However, resident *Wolbachia* infections in mosquitoes do not impact arboviral transmission to the same extent as transinfected *Drosophila Wolbachia* strains.

The epidemiology of JE would also be favorable for *Wolbachia-*based biocontrol. The generation and release of “JEV-refractory” *Cx*. *tritaeniorhynchus* could break the transmission cycle (Fig 1B) at various points. Firstly, the enzootic sylvatic cycle in reservoir bird hosts would be inhibited, preventing circulation of JEV in the local release area. In addition, the prevention of reservoir host bird infections would likely reduce the potential geographical expansion of JEV through bird migration. Secondly, *Wolbachia*-infected *Cx*. *tritaeniorhynchus* mosquitoes would inhibit the enzootic amplification cycle in pigs, significantly reducing overall transmission. Finally, the spillover of JEV transmission to humans would also be inhibited by the presence of *Wolbachia*-infected *Cx*. *tritaeniorhynchus*. As *Cx*. *tritaeniorhynchus* are highly zoophilic, outbreaks of JE occur when there is a rapid increase in mosquito populations resulting in a spillover of JEV from the enzootic animal host cycle to humans. Therefore, the potential increase in drought/flooding of rice fields due to changing agricultural practices and climate change in JEV endemic areas could lead to bursts of vector proliferation during flooding, resulting in greater outbreaks in the future [2]. Vector control strategies during outbreaks that target adult mosquitoes are often ineffective, as transmission is already occurring and space spraying does not effectively target *Cx*. *tritaeniorhynchus*. Alternative mosquito control strategies that aim to suppress the mosquito population, such as the sterile insect technique (SIT) [72,73] or release of insects with a dominant lethal (RIDL) [74,75], could have a potential role in JE control. However, the likely need for repeated release of large numbers of sterile males for individual outbreaks of JE over a large transmission area would also pose both logistical and financial difficulties. Therefore, a *Wolbachia*-based biocontrol strategy that aims to simply replace the existing vector population with mosquitoes that are unable to transmit JEV would likely prevent outbreaks occurring even when there is a rapid increase in mosquito vector population densities.

## Conclusions

Novel vector control methods for JE are needed, and *Wolbachia*-based biocontrol may provide sustainable, long-term control. The successful transinfection of *Drosophila Wolbachia* strains into *Cx*. *tritaeniorhynchus* is likely to result in JEV-refractory mosquito lines. The success of the first *Ae*. *aegypti* field trials in Australia indicates that a *Wolbachia*-based method of biocontrol is readily deployable in the field and also shows minimal environmental impact or safety concerns [76]. The stability of the arboviral blocking phenotype, in wild *Ae*. *aegypti* mosquitoes [59] and in the long-term evolutionary association between native *Wolbachia* strains in *Drosophila* flies [46], suggests an inhibitory effect on JEV with transinfected *Cx*. *tritaeniorhynchus* will be present for the medium to long term. If it is demonstrated that *Wolbachia*-infected JEV vectors cannot transmit the virus, it would suggest this biocontrol strategy could significantly reduce JE morbidity and mortality.

## Boxes

**Box 1. Key Learning Points**

- Vaccines for JE have been developed but are expensive, require multiple doses, and are unlikely to result in eradication, as humans are a “dead-end” host.
- Current vector control against the principal mosquito, *Cx*. *tritaeniorhynchus*, includes intermittent irrigation of rice fields and space spraying of insecticides during outbreaks.
- Significant advancements have been made in the potential use of the bacterial endosymbiont *Wolbachia* for mosquito biocontrol of DENV.
- *Cx*. *tritaeniorhynchus* do not harbor natural *Wolbachia* infections, and JEV is closely related to DENV (both in the *Flavivirus* genus).
- Successful establishment of *Wolbachia* strains in *Cx*. *tritaeniorhynchus* can be reasonably expected to significantly impact JEV transmission.

**Box 2. 5 Key Papers in the Field**

1. Erlanger TE, Weiss S, Keiser J, Utzinger J, Wiedenmayer K (2009) Past, Present, and Future of Japanese Encephalitis. Emerg Infect Dis 15: 1–7.
2. Solomon T, Ni H, Beasley DW, Ekkelenkamp M, Cardosa MJ, et al. (2003) Origin and Evolution of Japanese Encephalitis Virus in Southeast Asia. J Virol 77: 3091–3098.
3. Karunaratne SH, Hemingway J (2000) Insecticide Resistance Spectra and Resistance Mechanisms in Populations of Japanese Encephalitis Vector Mosquitoes, *Culex tritaeniorhynchus* and *Cx*. *gelidus*, in Sri Lanka. Med Vet Entomol 14: 430–436.
4. Walker T, Johnson PH, Moreira LA, Iturbe-Ormaetxe I, Frentiu FD, et al. (2011) The wMel *Wolbachia* Strain Blocks Dengue and Invades Caged *Aedes aegypti* Populations. Nature 476: 450–453.
5. Hoffmann AA, Montgomery BL, Popovici J, Iturbe-Ormaetxe I, Johnson PH, et al. (2011) Successful Establishment of *Wolbachia* in *Aedes* Populations to Suppress Dengue Transmission. Nature 476: 454–457.
